# *Clostridium thermocellum* ATCC27405 transcriptomic, metabolomic and proteomic profiles after ethanol stress

**DOI:** 10.1186/1471-2164-13-336

**Published:** 2012-07-23

**Authors:** Shihui Yang, Richard J Giannone, Lezlee Dice, Zamin K Yang, Nancy L Engle, Timothy J Tschaplinski, Robert L Hettich, Steven D Brown

**Affiliations:** 1 Biosciences Division, Oak Ridge National Laboratory, 1 Bethel Valley Road, Oak Ridge, Tennessee 37831, USA; 2 BioEnergy Science Center, Oak Ridge National Laboratory, 1 Bethel Valley Road, Oak Ridge, Tennessee 37831, USA; 3 Chemical Sciences Division, Oak Ridge National Laboratory, 1 Bethel Valley Road, Oak Ridge, Tennessee 37831, USA; 4 Present address: National Bioenergy Center, National Renewable Energy Laboratory, 15013 Denver West Parkway, Golden, Colorado 80401, USA

## Abstract

**Background:**

*Clostridium thermocellum* is a candidate consolidated bioprocessing biocatalyst, which is a microorganism that expresses enzymes for both cellulose hydrolysis and its fermentation to produce fuels such as lignocellulosic ethanol. However, *C. thermocellum* is relatively sensitive to ethanol compared to ethanologenic microorganisms such as yeast and *Zymomonas mobilis* that are used in industrial fermentations but do not possess native enzymes for industrial cellulose hydrolysis.

**Results:**

In this study, *C. thermocellum* was grown to mid-exponential phase and then treated with ethanol to a final concentration of 3.9 g/L to investigate its physiological and regulatory responses to ethanol stress. Samples were taken pre-shock and 2, 12, 30, 60, 120, and 240 min post-shock, and from untreated control fermentations for systems biology analyses. Cell growth was arrested by ethanol supplementation with intracellular accumulation of carbon sources such as cellobiose, and sugar phosphates, including fructose-6-phosphate and glucose-6-phosphate. The largest response of *C. thermocellum* to ethanol shock treatment was in genes and proteins related to nitrogen uptake and metabolism, which is likely important for redirecting the cells physiology to overcome inhibition and allow growth to resume.

**Conclusion:**

This study suggests possible avenues for metabolic engineering and provides comprehensive, integrated systems biology datasets that will be useful for future metabolic modeling and strain development endeavors.

## Background

*Clostridium thermocellum* is a Gram-positive, anaerobic, thermophilic bacterium that produces large extracellular enyzme complexes, called cellulosomes. It can rapidly solubilize biomass and it is a candidate microorganism for converting biomass into lignocellulosic ethanol via a process termed consolidated bioprocessing (CBP) (see reviews [1-5]. A targeted gene deletion system is a recent and important development for *C. thermocellum* studies and strain development [6,7], new genome sequences have become available [8] and fundamental studies have identified where large amounts of previously unaccounted for carbon is going during fermentations [9].

High product titer is an essential industrial consideration for capital and downstream processing costs [10]. *C. thermocellum* strains, such as SS22, have been selected for enhanced ethanol tolerance (64 g/L), and ethanol concentrations between 27 to 50 g/L only inhibited the growth of different strains (A1, C9, and S7) by approximately 50% [11]. However, increased ethanol tolerance and productivity are not necessarily linked, with the highest concentrations of ethanol produced reported as ≤ 30 g/L [12]. Membrane integrity has been recognized as a key factor in ethanol tolerance [13]. In addition, the role of compatible solutes such as trehalose, amino acids, such as proline [14,15], in ethanol tolerance and specific genes (e.g. [16-18]) have been investigated in a variety of microorganisms.

Differences in membrane proteins between *C. thermocellum* wild-type and ethanol-adapted (EA) strains have been investigated using proteomics [19]. Timmons et al. (2009) determined the fatty acid composition and membrane anisotropy from *C. thermocellum* wild-type and EA strains. They reported that EA had more fatty acids with chain lengths >16:0 and significantly more 16:0 plasmalogens compared to the parent and proposed a model that strain EA ethanol tolerance is due to fatty acid alterations that increase membrane rigidity and counter-act the fluidizing effect of ethanol [20].

Genome resequencing was conducted for *C. thermocellum* EA and a mutated alcohol dehydrogenase gene (*adhE*) with altered cofactor specificity was identified as a key genetic determinant for the enhanced ethanol tolerance phenotype [21]. Subsequently, two independent ethanol tolerant *C. thermocellum* mutants named E50A and E50C were selected using a strategy that alternated between increasing ethanol concentrations and transfers in medium that lacked selection pressure [22]. In contrast to strain EA, strains E50A and E50C grew as well as or better than the wild-type strain and similar to strain EA the mutations were identified in an alcohol dehydrogenase gene by resequencing. Common mutations were also identified in genes involved in arginine/pyrimidine biosynthetic pathways.

A detailed fundamental understanding of biological systems under standard and altered environmental conditions is important for metabolic engineering, synthetic biology, and to advance applied goals for enhanced biofuels production [23]. In this study, we combined transcriptomic, proteomic and metabolic profiling with bioinformatic analyses to elucidate the molecular responses of wild-type *C. thermocellum* to ethanol stress compared to untreated control samples. The combined approach, which employs three of the major “omic” technologies, endeavors to provide a deep and global insight into the molecular mechanisms of *C. thermocellum* ethanol stress responses. Though each analytical technology is powerful in its own right, the application of orthogonal “omic” measurements provides additional support for conclusions put forth in this study and provides avenues for future studies into the different aspects of physiology and regulation captured by the respective technologies.

## Methods

### Controlled batch fermentations

*C. thermocellum* ATCC27405 was cultured in chemically defined MTC medium with 5 g/L of cellobiose as the carbon source, as described previously [24,25] at 60°C. Batch fermentations were conducted in approximately 4.0 L of MTC medium in 7.5-L BioFlo110 bioreactors (New Brunswick Scientific, NJ) fitted with agitation, pH, and temperature probes and controls, as described previously [26]. Fermentors were sparged overnight with filter-sterilized N_2_ gas and for approximately one hour post-inoculation to maintain anaerobic conditions. The agitation rate was 300 rpm in each vessel. Culture pH was monitored using a pH electrode (Mettler-Toledo, Columbus, OH) and the pH control set point was maintained at pH 7.0 by automatic titration with 3 N NaOH or HCl. *C. thermocellum* was added to a serum bottle containing 50 mL MTC broth for inoculation. The optical density was measured with a spectrophotometer at 600_nm_ and the inoculum was added to one fermentor for the seed culture, and the culture from the seed culture fermentor was used to inoculate three fermentors to an initial OD600_nm_ of approximately 0.14. Two fermentations were conducted for the wild-type *C. thermocellum* controls (no ethanol supplementation) or 3.9 g/L (or 0.5% [v/v]) ethanol shock treatment. Growth was monitored turbidometrically by measuring optical density (OD) at 600_nm_ with a model 8453 spectrophotometer (Hewlett-Packard, CA.). Samples were harvested at approximately mid-exponential phase (OD 600_nm_ ~ 0.5) and at different time points post ethanol shock.

### Extracellular metabolite analysis with High-Performance Liquid Chromatography (HPLC)

HPLC analysis was used for the measurements of the extracellular metabolite concentration of cellobiose, acetate, and ethanol in 0.2 μm-filtered samples taken at different time points during fermentation, as described previously [26]. The fermentation samples were acidified with 10 mM sulfuric acid, separated and quantified by HPLC using a LaChrom Elite System (Hitachi High Technologies America, Inc., CA). Analysis was performed with the oven (Model L-2350) set at 60°C, and a pump (Model L-2130) set with a flow rate of 0.5 mL/min in 5 mM H_2_SO_4_. The run time for each sample was set for 35 min (Injector Model L-2200). Eluted compounds were registered and quantified by a refractive index detector (Model L-2490) equipped with a computer-powered integrator. Soluble fermentation products were identified by comparison with retention times and peak areas of corresponding standards. Metabolites were separated on an Aminex HPX-87 H, 300 x 7.8 mm column (Bio-Rad, CA).

### RNA extraction and ds-cDNA synthesis

RNA was isolated essentially as described previously [26], except that an additional bead beating step was included for efficient Gram-positive cell lysis. Briefly, samples were harvested by centrifugation, resuspended in TRIzol reagent (Invitrogen, CA) and subsequently 1.0 mL aliquots were transferred 2.0 mL screw cap tubes (#12800-200-E, MO BIO Laboratories, Inc., CA) that contained 0.25 g of lysis beads from a UltraClean Microbial RNA Kit (MO BIO Laboratories, Inc,). Cell lysis proceeded with 3 X 20 sec bead beating treatments at 6,500 rpm in a Precellys 24 high-throughput tissue homogenizer (Bertin Technologies, Montigny-le-Bretonneux, France). Cell lysates were transferred to fresh tubes and subsequently purified as described previously [26]. Each total RNA preparation was treated with RNase-free DNase I (Ambion, TX) to digest residual chromosomal DNA and subsequently purified using the RNeasy mini kit (Qiagen, CA). Total cellular RNA was quantified with a NanoDrop ND-1000 spectrophotometer (NanoDrop Technologies, DE) and RNA quality was assessed with Agilent Bioanalyzer (Agilent, CA). Purified RNA of high quality was used as the template to generate ds-cDNA using Invitrogen ds-cDNA synthesis kit (Invitrogen, CA).

### Microarray sample labeling, hybridization, scanning, and statistical analysis of array data

ds-cDNA was labeled, hybridized, arrays washed, and scanned following the NimbleGen protocols. Hybridizations were conducted using an 12-bay hybridization station (BioMicro Systems, Inc., UT), arrays dried using a Maui wash system (BioMicro Systems, Inc.) and scanned with a Surescan high-resolution DNA microarray scanner (Agilent Technologies, CA), and the images were quantified using NimbleScan software (Roche NimbleGen, IN). Statistical analyses were done with JMP Genomics 4.1 software (SAS Institute, NC), essentially as described previously [27]. The data were normalized using the LOWESS normalization algorithm within JMP Genomics. An analysis of variance (ANOVA) was performed to determine differential expression levels between conditions and time points using the False Discovery Rate (FDR) testing method (*p* < 0.05). Microarray data have been deposited in NCBI Gene Expression Omnibus (GEO) database under accession number GSE25236. The interactions among differentially expressed genes were investigated using the String 8.2 pre-computed database [28], available at http://string.embl.de/.

### Quantitative-PCR (qPCR) analysis

Microarray data were validated using real-time qPCR, as described previously [26,27], except that the Bio-Rad MyiQ2 Two-Color Real-Time PCR Detection System (Bio-Rad Laboratories, CA) and Roche FastStart SYBR Green Master (Roche Applied Science, IN) were used for this experiment. Eleven genes representing different functional categories and range of gene expression values based on microarray hybridizations were analyzed using qPCR from cDNA derived from different time point samples. Oligonucleotide sequences of the eleven genes selected for qPCR analysis are listed in Additional file 1.

### Intracellular metabolite analysis with gas chromatography–mass spectrometry (GC-MS)

Culture samples were rapidly pelleted by centrifugation, supernatants removed, cell pellets snap-frozen in liquid nitrogen and then stored at −80°C until analysis. Krall et al. (2009) conducted a rigorous comparison of sampling approaches for microbial cultures and concluded that fast filtering and centrifugation (even at room temperature) produced similar concentrations of metabolites, even for those predicted with high turnover [29]. Metabolite analyses were performed on microbial pellets collected in different time points suspended with 80% ethanol (aqueous), as described previously [26]. Briefly, cells were disrupted using a sonicator 3000 (Misonix, Inc., NY). An internal standard of 200 μL of sorbitol (1 mg/mL aqueous solution) was then added to each tube and 2-mL aliquots then dried in a helium stream. Metabolites were converted to trimethylsilyl derivatives and analyzed with an Agilent Technologies Inc. (CA) 5975 C inert XL gas chromatograph-mass spectrometer, fitted with an HP-5MS (5% Phenyl Methyl Silox) 30 m x 250 μm x 0.25 μm film thickness capillary column, as described previously [30]. Two biological samples from each condition were analyzed with metabolite data of *C. thermocellum* under control and ethanol treatment conditions averaged and presented as relative responses between *C. thermocellum* under ethanol treatment versus *C. thermocellum* without ethanol treatment fermentation. Statistically significant treatment differences were determined by Students t-tests with probability values *p* < 0.10 shown.

### Sample preparation for 2D-LC-MS/MS

*C. thermocellum* pelleted cells from time point 120 min were prepared for LC-MS analysis as follows. Cells were lysed by a combination of SDS (4% SDS in 100 mM Tris–HCl pH 8.0), sonic disruption at 20% amplitude (Branson Digital Sonifier), and boiling, followed by centrifugation at 21000 *g* for 10 min. Crude lysates were assayed for protein concentration via bicinchoninic acid assay (BCA), (Pierce, IL), adjusted to 50 mM dithiothreitol (DTT), and incubated at 60°C for 10 min. Trichloroacetic acid (TCA) precipitation of 2 mg of crude protein was performed to remove bulk SDS and other small molecules that could potentially interfere with downstream MS measurement. Pelleted protein was washed twice with ice-cold acetone, air-dried to remove residual acetone, and resuspended in 250 μL of denaturation buffer (8 M urea, 100 mM Tris–HCl pH 8.0). Denatured proteins were then reduced with 5 mM DTT for 10 min at room temperature (RT), alkylated with 10 mM iodoacetamide (IAA) for 10 min at RT in the dark, and diluted to 4 M urea for tryptic digestion. Proteins were digested overnight at a 1:100 trypsin to protein ratio (w/w), and again in 2 M urea for 4 h the following day. Resulting peptides were then adjusted to 150 mM NaCl, 0.1% formic acid, filtered through a 10 kDa spin column filter (VWR, PA), and quantified by BCA for subsequent LC-MS analysis.

### Multi-dimensional LC-MS analysis of peptides

Peptide samples were directly loaded onto a biphasic MudPIT back column packed with 4 cm strong-cation exchange (SCX) resin followed by 3 cm C18 reversed phase (RP) resin (Luna and Aqua respectively, Phenomenex), as previously described [31,32]. Adsorbed peptides were washed offline with solvent A (5% acetonitrile, 95% HPLC-grade water, 0.1% formic acid) for 20 min, followed by a 25 min gradient to solvent B (70% acetonitrile, 30% HPLC-grade water, 0.1% formic acid). The back column containing the peptides was placed in-line with an LTQ-XL MS (Thermo Fisher Scientific Inc., MA) outfitted with an in-house pulled nanospray emitter (100 micron ID) packed with 15 cm of C18 RP material. Peptides were separated in two chromatographic dimensions (charge and hydrophobicity) over an 11-step MudPIT analysis (salt pulses [5%, 7.5%, 10%, 12.5%, 15%, 17.5%, 20%, 25%, 35%, 50%, and 100% of 500 mM ammonium acetate] each followed by a 2 h organic gradient to 50% solvent B). Tandem mass spectra (2 μscans) were acquired in data-dependent mode based on full-range mass scans (2 μscans). Two technical replicates were analyzed per sample.

### Database searching and semi-quantitative proteomics data analysis

Tandem mass spectra collected for each sample were matched to specific peptide sequences via SEQUEST [33] utilizing a FASTA protein database containing *C. thermocellum* (NC_009012, version 08-FEB-2007) and common contaminant protein entries, as well as reversed decoy sequences to assess false-discovery rates. As IAA was used to block the reformation of disulfide linkages, a static modification present at all cysteine residues (carboxymethylation, +57 Da) was utilized. SEQUEST-derived peptide sequence data were then filtered with DTASelect [34] and assembled into protein loci using the following score thresholds: XCorr: +1 = 1.8, +2 = 2.5, +3 = 3.5, DeltCN 0.08, and 2 peptides (1 unique) per protein identification. These criteria were chosen to produce average protein-level FDRs between 1 and 2 percent. DTASelect-filtered data for each sample run were then prepared for semi-quantitative analysis. Before calculation of normalized spectral abundance factors (NSAF) [35], spectral counts (SpC) of non-unique/redundant peptides were weighted based upon the relative abundance of the proteins that share these sequences, as derived by their total unique SpC. Once rebalanced, NSAF values were calculated for each protein and imported into JMP Genomics 4.1 (SAS, Inc., NC) for data normalization, clustering, and significance testing. Proteomics data can be accessed at: https://compbio.ornl.gov/mspipeline/besc/Ctherm_EtOH_Stress/analysis/Brown/mzML/Ctherm_EtOH_Stress_Raw_Data.tar.gz and https://compbio.ornl.gov/mspipeline/besc/Ctherm_EtOH_Stress/analysis/Brown/mzML/Ctherm_EtOH_Stress_SEQ_DTA_Data.tar.gz.

## Results

The time point in mid-exponential growth at which ethanol was added into the treatment fermentors was designated as “ethanol-shock” or “time zero”. Samples are either referred to as “control” for untreated control fermentations or “treatment” or “ethanol treatment” for those derived from fermentations that received 3.9 g/L (0.5% < v/v>) ethanol at “time zero”.

### *C. thermocellum* growth response to ethanol and extracellular metabolite profiles

**Figure 1:**
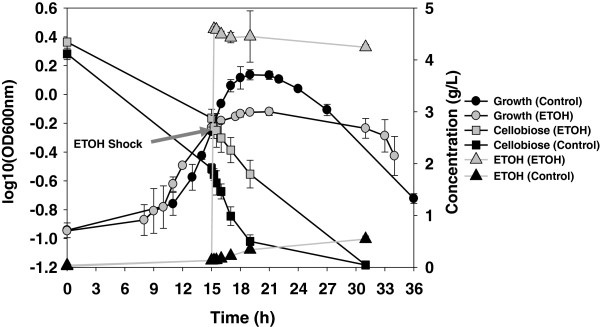
***C. thermocellum *growth, cellobiose consumption and ethanol production in the absence or presence of 3.95 g/L (or 0.5% [v/v]) ethanol.** Arrow indicates when ethanol was added to the treatment fermentors. Samples were collected at time zero and 2, 12, 30, 60, 120, 240 min after ethanol supplementation. Final time point for control fermentors is 36 h and 33 h for treatment fermentors.

Culture turbidity, as measured by OD_600_ units, for the ethanol-treatment fermentors prior to ethanol shock was similar to the control fermentors (0.59 ± 0.02 vs 0.55 ± 0.02, respectively) (Figure 1). The culture densities for both the treatment and control conditions increased during the “omics” sampling period (2 h post ethanol addition), however the supplementation of ethanol negatively influenced *C. thermocellum* growth and cellobiose consumption (Figure 1). The maximal culture density over the experiment for the control fermentors was OD_600_ 1.37 ± 0.11, which occurred 4 h after time zero. In contrast, the maximal cell density for the treatment fermentors was OD_600_ 0.76 ± 0.04, 6 h post ethanol treatment. The culture density for both the treatment and control decreased as cells entered late stationary phase.

HPLC was used to quantify and compare the kinetics of extracellular substrate (cellobiose) consumption, and extracellular end-product (ethanol, acetate, and lactate) formation. The final cellobiose concentrations were similar between the ethanol-treated and control cells (Figure 1). However, the cellobiose consumption rate was reduced from 0.46 g/L/h in the absence of ethanol to 0.24 g/L/h after ethanol treatment. Ethanol production was correlated with the cellobiose consumption (Figure 1). At the conclusion of the experiment the ethanol concentration in control fermentations was approximately 0.41 ± 0.08 g/L. In contrast, ethanol supplementation halted its production in the treatment fermentors and there was a slight decrease in net ethanol concentration to about 0.35 ± 0.013 g/L after ethanol treatment. In addition, the acetate production rate was reduced by half, from 0.10 g/L/h to 0.05 g/L/h, with the supplementation of ethanol. However, the final concentration of acetate was slightly higher in the ethanol-treated cells (1.00 ± 0.03 g/L) than that of control cells (0.87 ± 0.06 g/L). Lactate was also produced during the experiment, with the majority being produced after cellobiose was largely consumed. The highest lactate concentration was found in the ethanol-treated cells (0.035 ± 0.016 g/L), compared to 0.014 ± 0.003 g/L for the control cells.

### Intracellular metabolomic profiles reveals a reduction in glutamic acid and accumulation of sugar phosphates

The physiological status of *C. thermocellum* was investigated further by GC-MS analysis of the intracellular metabolomic profiles from time zero and at different time points post ethanol-shock (2, 12, 30, 60, 120, 240 min). Metabolomic analyses indicated that there were few relative metabolite responses (*C. thermocellum* ethanol-treated versus control cells) that were significantly different at different time points with the number of replicates used in this study (Additional file 2). Ethanol-treated cells indicated a decline in glutamic acid, with it being reduced to 19% of the controls as early as 30 min after ethanol shock. The only other significant response in an amino acid was a 2.8-fold increase in phenylalanine within the ethanol-treated cells that occurred at 240 min (Additional file 2). These responses were accompanied by a doubling of sugar phosphates, including glucose-6-phosphate and fructose-6-phosphate that were significant at 60 and 120 min post treatment, whereas glucose-1-phosphate was unaffected (Additional file 2). The substrate cellobiose tended to be relatively higher in the ethanol-treated cells. The only other significant response was a 1.2-fold increase in 3-phosphoglyceric acid in the ethanol-stressed *C. thermocellum* compared to that of untreated cells; accumulating at 240 min, but was not significantly different earlier (Additional file 2).

### Transcriptomic profile of *C. thermocellum* in response to ethanol

#### Global view of ethanol response

**Figure 2:**
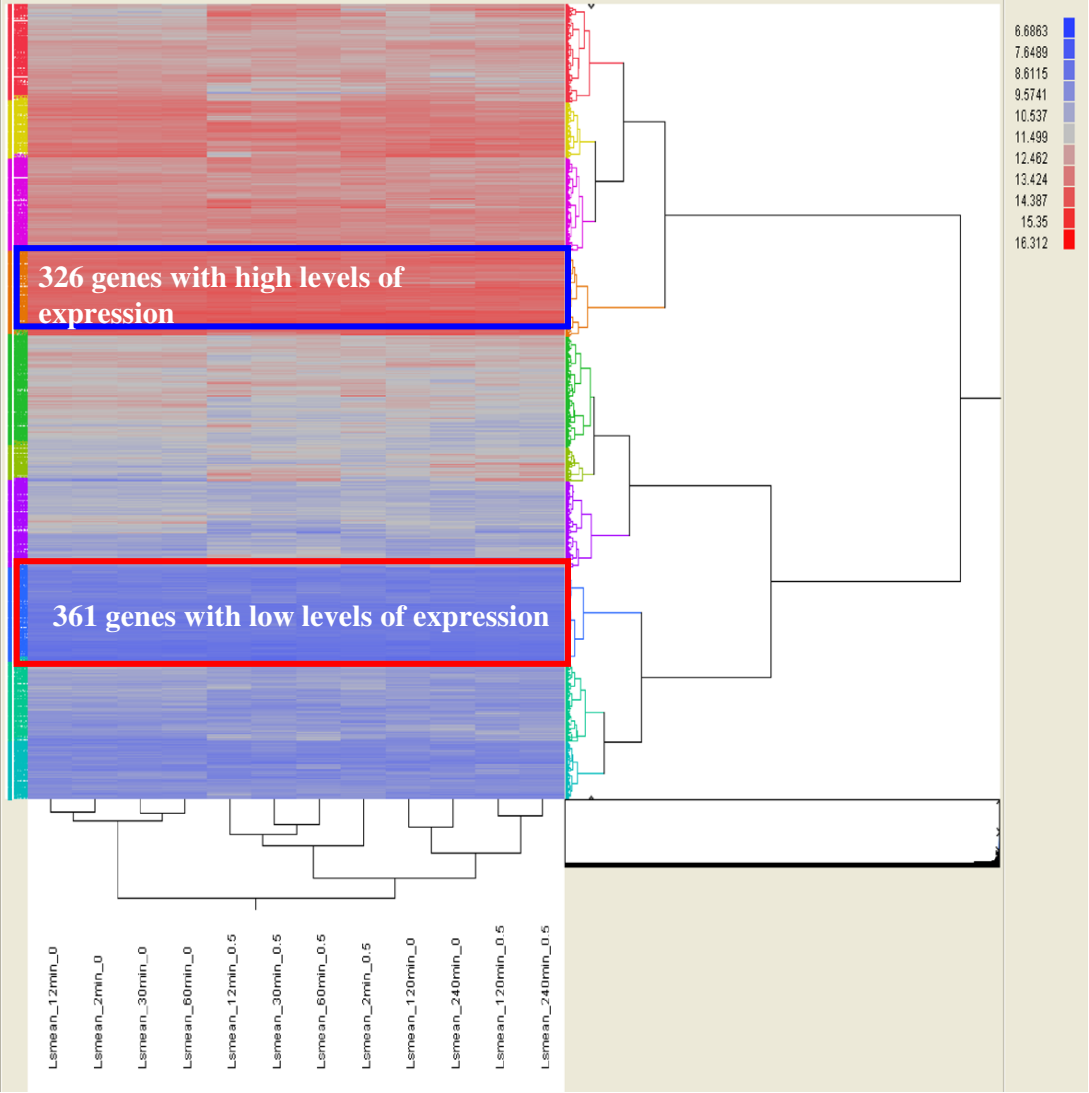
**Overview of ethanol shock expression responses by hierarchical clustering.** Microarray probe intensity values (log_2_ transformed) for treatment and control arrays were clustered using JMP Genomics. Details for gene expression values can be found in Additional file 3.

Gene expression profiles for the control and ethanol-treated fermentations were generated from samples harvested at time zero, 2, 12, 30, 60, 120, and 240 min post ethanol-shock using NimbleGen microarrays. We identified significantly differentially expressed genes by comparing the gene expression profiles of treated cells to that of control cells at the same time points, as well as the time-course gene expressions for control cells or ethanol-treated cells separately. In the absence of fold-change filtering, more than three thousand genes were significantly differentially expressed in at least one of the multiple comparisons covering nearly all 3,198 predicted *C. thermocellum* genes, (Figure 2, Additional file 3), which is consistent with a recent report that detected expression for 2,846 *C. thermocellum* genes when the cells were grown in either cellulose or cellobiose limited chemostats [36].

**Figure 3:**
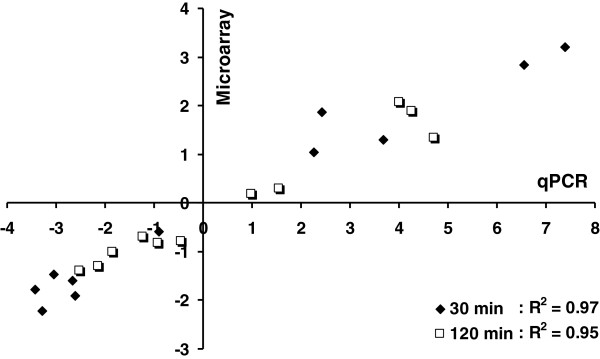
**Correlation between microarray and real-time qRT-PCR results for microarray data verification.** Comparison of gene expression measurements by microarray and qRT-PCR between *C. thermocellum* with the treatment of 3.95 g/L ethanol and control cells without ethanol treatment at 30 and 120 min post ethanol-shock. The gene expression ratios of both microarray data and qPCR data were log transformed in base 2 (log_2_ < Ethanol_treatment/Control>), and the microarray log2 ratio values were plotted against the qRT-PCR log2 values. The eleven genes are Cthe_0197, Cthe_0665, Cthe_0736, Cthe_0745, Cthe_1028, Cthe_1539, Cthe_1565, Cthe_2301, Cthe_2336, Cthe_2435, and Cthe_3016.

Eleven differentially-expressed genes from the treatment versus control comparison representing different functional categories and a broad expression range were selected for real-time quantitative PCR (qPCR) confirmation (Figure 3, Additional file 1, and 3). Correlation coefficient values of *R*^*2*^ = 0.97 and 0.95 were obtained for comparisons between microarray and qPCR for the time points of 30 and 120 min, respectively (Figure 3), which indicated microarray data were of good quality.

In this study, 326 genes showed high levels of expression with normalized expression intensity values ≥ 4000 (arbitrary units) and 361 genes with relatively low expression intensity (<1000) across all conditions (Figure 2, Additional file 3). Many phage- or sporulation-related genes and those with unknown function were among genes that had low expression levels (Additional file 3). In contrast, genes that were expressed at higher levels were related to cellulosome components, protein synthesis including ribosomal proteins, electron transfer components, and energy metabolism and a small portion of genes of unknown function were also highly expressed (Additional file 3). Nearly all the 326 protein-coding genes in the abundant subset were represented in the proteome, with only 29 that were not detected at 120 min post-shock (Additional files 3 and 4). In contrast, only 7 proteins of the 361 protein-coding genes expressed at low levels were detected (Additional files 3 and 4).

#### Ethanol responsive genes based on treatment versus control comparisons

**Figure 4:**
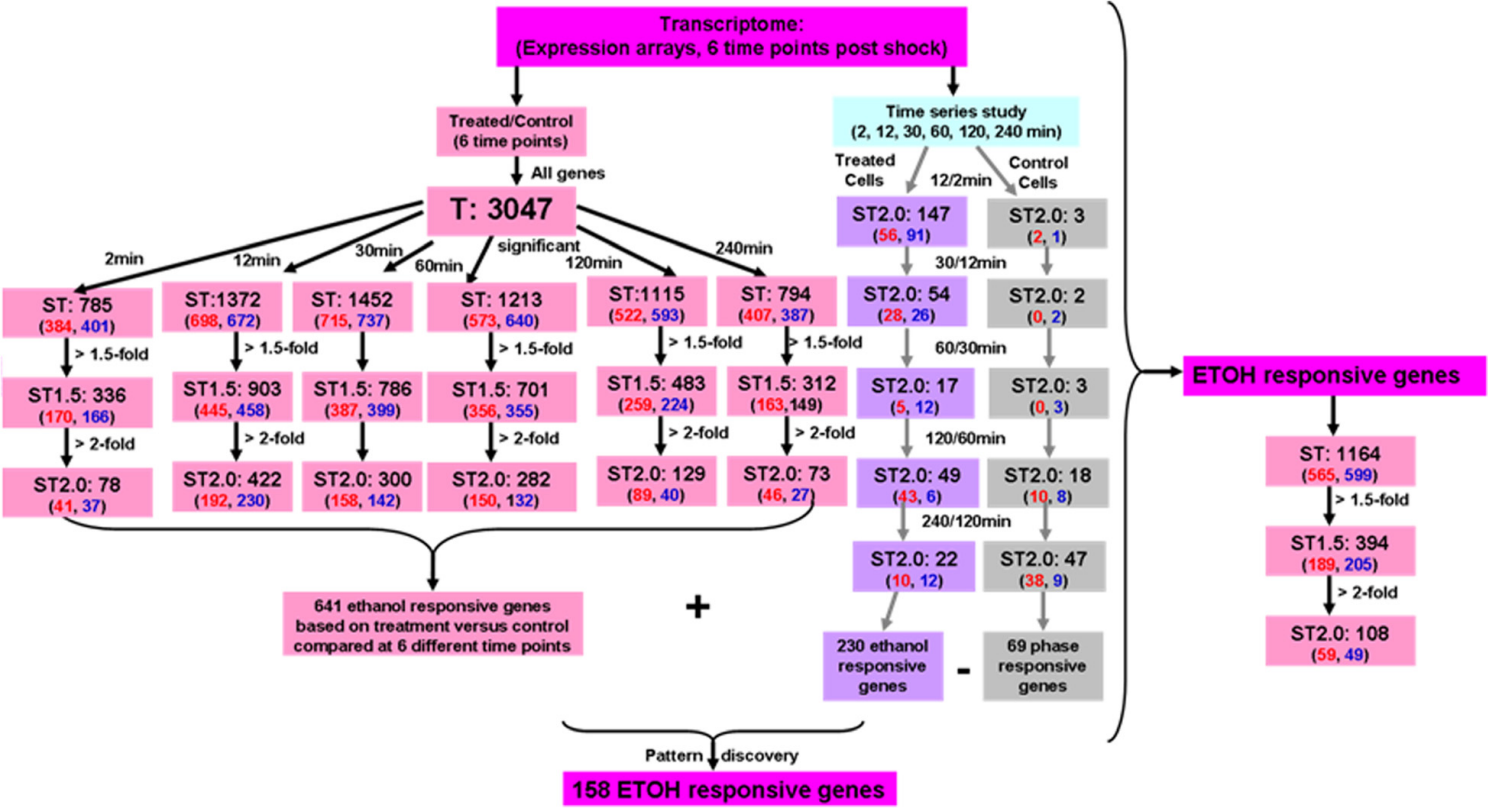
**Flowchart of gene numbers for different transcriptome comparisons used to identify ethanol-responsive genes.** Samples were taken at different time of 2, 12, 30, 60, 120, and 240 min after ethanol shock. Six time point comparisons of ethanol treated cell versus control cells as well as time course comparison for control cells or ethanol-treated cells were done. T: all the genes identified from transcriptomics; ST: significant genes; ST1.5: significant genes with at least 1.5-fold changes; ST2.0: significant genes with at least 2-fold changes. The numbers after above symbols are the total number identified, and the numbers underneath the symbols are ethanol up-regulated with red color font followed by ethanol down-regulated with blue font.

When ethanol-treated cells were compared to control cells at the same time points, i.e. 2 min (time zero untreated control was compared to 2 min post-shock), 12, 30, 60, 120, and 240 min, 641 genes were significantly differentially expressed with at least a 2-fold change (Figure 4, Additional file 5). Immediately after ethanol-shock, the cells responded promptly by regulating their gene expression such that after only 2 min, 78 genes were significantly differentially expressed with at least a 2-fold change (Figure 4, Additional file 5). At 12 min post ethanol-shock, expression levels for nearly 13% of the predicted genes were significantly different, while at 30 and 60 min post-shock about 300 genes (~9%) were differentially expressed (Figure 4, Additional file 5). The number of genes expressed differently between ethanol-treated cells and control cells was reduced when the cells began to adapt to the ethanol stress condition. There were 129 and 73 differentially expressed genes at the time points of 120 and 240 min post ethanol-shock, respectively (Figure 4, Additional file 5). A *C. thermocellum* alcohol dehydrogenase (*adhE*) with altered cofactor specificity has been identified as a key genetic determinant for enhanced ethanol tolerance in a mutant strain [21]. In this wild-type study, differential *adhE* expression was not a predominant response and cognate treatment/control differences were always < 2 fold (Additional file 3), supporting the earlier study that indicated a change in AdhE co-factor specificity was important for ethanol tolerance rather than the *adhE* over-expression [21].

#### The dynamics of ethanol responsive gene expression

Out of the 641 significantly differentially expressed ethanol-responsive genes (Additional file 5), approximately half were up-regulated and approximately half were down-regulated ≥ 2-fold in at least one time point comparison. An overview of how different comparisons and the effect of using different fold change stringency is presented (Figure 4). Nine genes were constantly up-regulated and three genes were constantly down-regulated at least 2-fold (Additional file 5). The nine genes constitutively up-regulated with ethanol treatment had functions in amino acid transport and metabolism, including glutamine synthetase catalytic region (Cthe_0196), glutamine amidotransferase (Cthe_0197), urease gamma subunit (Cthe_1818), diaminopimelate epimerase (Cthe_3100), and a predicted urea ABC transporter operon containing five genes from Cthe_1819 to Cthe_1823 (Additional file 5). Another seven genes were also constitutively up-regulated by ethanol treatment, except their ratios were less than 2-fold when compared to the earliest time point of 2 min (Additional file 5). However, some of them clustered together and belong to same predicted operons of nine constitutively up-regulated genes as discussed above (Additional file 5). These seven genes were therefore included as constitutive ethanol up-regulated genes. The three constitutively ethanol down-regulated genes include a hypothetical protein-signal peptide (Cthe_0746) and two genes belonging to the Ech hydrogenase operon (Cthe_3022 and Cthe_3023). Another gene (Cthe_3024) in this predicted operon was also down-regulated at all time points, but at time points 120 and 240 min log_2_ based ratio differences were −0.53 and −0.71, respectively, and these values did not meet the stringent criteria (Additional file 5). A predicted operon adjacent to the hypothetical gene Cthe_0746 contained five genes (from Cthe_0747 to Cthe_0751) related to spermidine/putrescine ABC transporter was also constitutively down-regulated except that the ratios were less than 2-fold at only one time point (Additional file 5).

#### Ethanol responsive genes based on time course comparison and their interactions

Temporal gene expression between the control or treatment condition were used to identify the ethanol responsive genes and exclude those that may be more related to growth-phase. These are presented as supplemental data and comprise of 158 core ethanol-responsive genes (Figure 4, Additional file 6).

**Figure 5:**
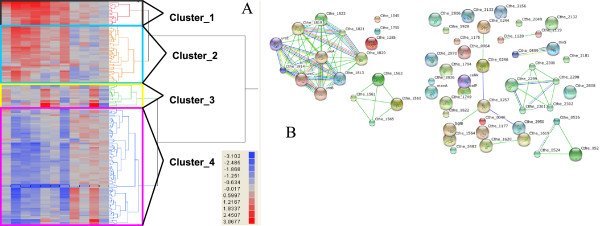
**Hierarchical clustering (A), and pre-existing interaction (B) analyses of the 158 ethanol-responsive genes.** Details for different clusters can be found in Additional file 6 and clustering was conducted using JMP Genomics.

A hierarchical clustering analysis of these 158 core ethanol-responsive genes partitioned the temporal profiles into four clusters, each of which were further analyzed using the STRINGS 8.2 database of pre-existing interactions [28] (Figure 5; Additional file 6). The first cluster (Cluster 1) included 17 genes, with the majority of these constitutively up-regulated with ethanol treatment (Figure 5; Additional file 6). Cluster 2 contained 41 genes that were mostly up-regulated, especially in earlier time points after ethanol treatment. Cluster 3 was represented by 17 genes that were regulated dynamically across the post-shock time course and Cluster 4 had 83 genes that were down-regulated upon ethanol treatment. The 17 genes within cluster 1 encode products related to urea utilization and likely its uptake, as well as a cysteine synthase homolog (Cthe_1560) and a nitrogenase homolog (Cthe_1565), and contain most of the genes for five predicted operons (Figure 5B) [37]. Cluster 2 contains genes related to CRISPR and other hypothetical functions (Figure 5B). Most gene products in Cluster 3 are likely involved in substrate translocation and amino acid biosynthesis (Figure 5B). Ethanol down-regulated genes in Cluster 4 are related to energy metabolism, and include genes for hydrogeneases, electron transport, cellulosome components, ribosomal protein synthesis, and a member of the LacI family of transcriptional regulators, amongst others. (Figure 5B). In summary, expression of genes participating in amino acid transport and metabolism were induced by ethanol, whereas the ethanol down-regulated genes primarily belonged to the categories of translation, ribosomal structure and biogenesis (Figure 5; Additional file 3).

### Proteomic profile of *C. thermocellum* in response to ethanol

Shotgun proteomics was carried out using the 120 min control and treated cell samples to measure the terminal time point in the ethanol stress/control comparison and to ascertain the overall impact on the final set of proteins and enzymes. More than thirteen hundred proteins were identified, which represents the largest number of *C. thermocellum* proteins detected to date (Additional file 4). Of these, 77 proteins exhibited a ≥ 1.5-fold and significant change (*p* ≤ 0.05) in abundance (Table 1). Forty-two proteins were more abundant within ethanol-treated cells, with three specific to only ethanol-treated cells at the 120 min time point (Table 1). The corresponding gene expression profiles for ethanol-specific proteins were also found to be significantly up-regulated (≥1.5-fold) and included three genes (Cthe_0197, 0198, 0200) belonging to a predicted seven gene operon related to nitrogen metabolism (Table 1). Ethanol up-regulated proteins also included several other proteins related to nitrogen metabolism, such as glutamine synthetase (Cthe_0196 and Cthe_1539).

**Table 1:** *C. thermocellum* proteins different between ethanol treatment and control

**Gene**	**Product**	**Protein relative fold change (Treatment/Control)**	**Array relative fold change (Treatment/Control)**	**Proteomics p-value**	**Array p-value**	**Array Sig Index**
Cthe_0042	small GTP-binding protein	1.8	1.6	5.0E-03	1.1E-02	1
Cthe_0076	hypothetical protein	1.5	1.2	5.0E-02	3.3E-01	0
Cthe_0087	maf protein	0.04	0.9	2.0E-02	3.2E-01	0
Cthe_0129	metal dependent phosphohydrolase	0.58	1.3	4.0E-03	9.1E-07	1
Cthe_0196	glutamine synthetase, catalytic region	2.4	2.5	2.0E-05	3.0E-11	1
Cthe_0197	glutamine amidotransferase, class-II	38	4.3	4.0E-07	1.5E-14	1
Cthe_0198	Glutamate synthase (NADPH)	7.6	4.1	1.0E-02	3.5E-14	1
Cthe_0200	FAD-dependent pyridine nucleotide-disulphide oxidoreductase	46	1.4	2.0E-02	3.4E-05	1
Cthe_0266	methyl-accepting chemotaxis sensory transducer	0.66	1	2.0E-02	9.8E-01	0
Cthe_0362	transcriptional regulator, AsnC family	0.64	1	7.0E-03	7.8E-01	0
Cthe_0363	aminotransferase, class I and II	0.64	0.9	9.0E-03	4.9E-01	0
Cthe_0395	RbsD or FucU transport	0.02	0.8	2.0E-02	1.9E-02	1
Cthe_0402	copper amine oxidase-like protein	1.6	1.3	2.0E-03	1.3E-03	1
Cthe_0550	heat shock protein Hsp90	0.62	1.1	2.0E-02	6.9E-01	0
Cthe_0559	single-strand binding protein/Primosomal replication protein n	0.33	1.1	2.0E-02	3.0E-01	0
Cthe_0567	peptide deformylase	0.59	1.3	2.0E-02	3.1E-04	1
Cthe_0626	hypothetical protein	0.62	1.1	2.0E-02	1.9E-02	1
Cthe_0681	IMP dehydrogenase/GMP reductase	0.63	1	2.0E-02	3.4E-01	0
Cthe_0715	Adenosylmethionine decarboxylase	0.02	0.5	4.0E-04	7.6E-04	1
Cthe_0849	3-dehydroquinate dehydratase, type II	4.6	2	3.0E-02	3.1E-16	1
Cthe_0902	3-methyl-2-oxobutanoate hydroxymethyltransferase	1.7	1.2	2.0E-03	2.7E-03	1
Cthe_0904	protein-export membrane protein SecD	0.62	0.8	2.0E-02	1.8E-04	1
Cthe_0949	carbamoyl-phosphate synthase, large subunit	1.8	0.7	2.0E-04	1.3E-07	1
Cthe_0951	orotidine 5'-phosphate decarboxylase	1.8	0.7	1.0E-02	1.8E-04	1
Cthe_0953	aspartate carbamoyltransferase	1.9	0.6	1.0E-02	2.0E-02	1
Cthe_0954	Uracil phosphoribosyltransferase	1.5	0.9	4.0E-02	3.5E-02	0
Cthe_0961	aspartate-semialdehyde dehydrogenase	1.8	1.2	3.0E-04	1.1E-03	1
Cthe_1102	fimbrial assembly protein	0.35	1.1	3.0E-02	2.3E-01	0
Cthe_1104	prepilin-type cleavage/methylation	0.61	1	2.0E-02	7.8E-01	0
Cthe_1165	YbbR-like protein	0.49	0.8	6.0E-03	9.5E-02	0
Cthe_1178	isochorismatase hydrolase	1.7	1.6	1.0E-02	1.3E-02	1
Cthe_1212	hypothetical protein	1.8	0.7	9.0E-03	1.0E-05	1
Cthe_1222	RNA methyltransferase, TrmH family, group 3	2.7	1.2	3.0E-02	4.8E-01	0
Cthe_1286	peptidase S1 and S6, chymotrypsin/Hap	2.1	1.1	2.0E-03	6.6E-02	0
Cthe_1326	small GTP-binding protein	0.48	1.1	8.0E-03	2.2E-01	0
Cthe_1329	putative CoA-substrate-specific enzyme activase	0.62	0.9	5.0E-03	1.9E-01	0
Cthe_1350	single-strand binding protein	0.49	0.6	2.0E-02	1.8E-05	1
Cthe_1383	Tetratricopeptide TPR_2	7	0.9	5.0E-03	4.2E-02	0
Cthe_1391	2-isopropylmalate synthase	2.4	0.6	3.0E-03	7.1E-17	1
Cthe_1433	short-chain dehydrogenase/reductase SDR	1.7	1	5.0E-03	3.0E-01	0
Cthe_1539	glutamine synthetase, catalytic region	2.4	3.7	4.0E-03	1.5E-25	1
Cthe_1556	binding-protein-dependent transport systems inner membrane component	0.26	1.2	4.0E-02	1.1E-04	1
Cthe_1570	extracellular solute-binding protein, family 3	0.59	1.6	1.0E-02	7.1E-05	1
Cthe_1605	phosphate ABC transporter (binding protein)-like protein	1.7	1.1	6.0E-03	7.1E-01	0
Cthe_1773	peptidase S16, lon-like protein	0.14	1	6.0E-03	9.3E-01	0
Cthe_1778	copper amine oxidase-like protein	5.1	1.5	2.0E-02	7.1E-03	1
Cthe_1801	ABC transporter related protein	1.9	0.5	8.0E-03	5.0E-15	1
Cthe_1823	Extracellular ligand-binding receptor	49	6.2	1.0E-07	2.5E-28	1
Cthe_1844	transcriptional regulator, BadM/Rrf2 family	0.22	1.2	3.0E-02	1.8E-03	1
Cthe_1911	Carbohydrate binding family 6	2.1	1.1	4.0E-02	1.9E-01	0
Cthe_1912	copper amine oxidase-like protein	1.6	0.9	2.0E-02	6.0E-01	0
Cthe_1922	hypothetical protein	1.7	1.1	5.0E-02	6.9E-01	0
Cthe_1955	RNA binding S1	0.6	1	4.0E-02	7.1E-01	0
Cthe_2095	hydrolase, TatD family	1.6	0.7	5.0E-02	6.8E-07	1
Cthe_2166	putative PAS/PAC sensor protein	0.2	0.7	1.0E-02	2.1E-08	1
Cthe_2171	type III restriction enzyme, res subunit	0.62	1.5	6.0E-03	6.6E-07	1
Cthe_2333	two component transcriptional regulator, winged helix family	0.55	0.9	3.0E-02	4.9E-01	0
Cthe_2403	4-diphosphocytidyl-2 C-methyl-D-erythritol kinase	0.65	1.4	2.0E-02	2.4E-08	1
Cthe_2423	hypothetical protein	2.9	0.9	1.0E-03	7.6E-01	0
Cthe_2424	copper amine oxidase-like protein	3.3	1	6.0E-03	8.9E-01	0
Cthe_2517	acetolactate synthase, small subunit	0.62	0.9	3.0E-02	2.0E-02	1
Cthe_2531	sulfate ABC transporter, periplasmic sulfate-binding protein	1.6	4.4	2.0E-02	1.0E-11	1
Cthe_2706	ABC transporter related protein	1.5	1.2	3.0E-02	4.3E-02	0
Cthe_2819	methyl-accepting chemotaxis sensory transducer	0.21	0.7	2.0E-02	7.2E-06	1
Cthe_2875	sigma 54 modulation protein/ribosomal protein S30EA	1.5	1	9.0E-03	7.9E-01	0
Cthe_2880	histidyl-tRNA synthetase	2.5	1.1	3.0E-03	8.1E-01	0
Cthe_2882	Histidinol dehydrogenase	1.7	0.8	1.0E-02	4.1E-02	0
Cthe_2883	histidinol-phosphate aminotransferase	1.9	0.8	2.0E-04	1.4E-02	1
Cthe_2889	phosphoribosyl-AMP cyclohydrolase	1.8	1.1	1.0E-02	3.2E-01	0
Cthe_2907	ribosomal protein S19	0.65	0.8	3.0E-02	2.5E-02	0
Cthe_3062	signal transduction histidine kinase regulating citrate/malate metabolism	0.63	0.8	3.0E-02	5.4E-03	1
Cthe_3076	Radical SAM	0.45	0.9	8.0E-03	4.7E-01	0
Cthe_3100	Diaminopimelate epimerase	1.6	3.3	1.0E-02	2.1E-07	1
Cthe_3101	aminotransferase, class I and II	1.8	2.2	2.0E-02	3.7E-06	1
Cthe_3107	Radical SAM	0.59	1.4	4.0E-02	2.0E-02	1
Cthe_3157	pyruvate carboxyltransferase	1.5	1.9	3.0E-03	3.0E-17	1
Cthe_3183	TrkA-N	0.04	0.9	2.0E-02	3.6E-01	0

Array significance determined by *p*-value (p < 0.05) and a significant array different is represented by a 1 in the Array Sig Index column and a 0 is not considered significantly different for a given gene at this time point comparison.

Thirty-five down-regulated proteins following ethanol treatment included three that were specific to the control, but only one of these (Cthe_0395) trended similar to the microarray data (≥1.5-fold change) (Table 1). Of the other 32 ethanol down-regulated proteins, only one exhibited a change (≥1.5-fold) in gene expression (single-strand binding protein, Cthe_1350) (Table 1).

AdhE was detected and it showed significant upregulation (*p* = 0.028) after treatment, but only at low levels (1.4 fold) (Additional file 4). In general, smaller differences in relative protein abundance levels were observed for proteins that were more prevalent within the cells, compared to those that had few spectra normalized counts (nSpC) (Additional file 4). A bivariate fit of relative fold changes (Treatment/Control) for the 120 min proteomics and transcriptomics profiles generated a correlation coefficient of *R*^*2*^ = 0.34.

### Ethanol effect on expression for cellulosome components

There are greater than 70 *C. thermocellum* cellulosome related proteins [38-40] (Additional file 7). Microarray results indicate that cellulosome genes were up-regulated, especially in the earlier time points (before 1 h) and that the most dramatic changes occurred within 12 min post-shock (Additional file 3 and Additional file 7). At 120 min post-shock, about forty cellulosome proteins were detected by proteomics (Additional file 5), and none of these proteins were identified as differentially expressed. Hierarchical clustering identified thirteen cellulosome component genes (e.g. *cipA, olpA,* and *celA, B, F, G, K, S*) that had high levels of constitutive expression for both control and treatment conditions, and similarly high protein abundances (Additional file 7). In the case of *cipA* and *celS*, recent genetic studies have affirmed their importance in cellulose degradation [6,41] and they are among the most highly *C. thermocellum* expressed genes with cellulose or cellobiose substrates in chemostat culture [36].

Twelve cellulosome related genes with low levels of gene expression were not detected via proteomics, and these are primarily dockerin components. In addition, within the 158 ethanol-responsive genes, six cellulosome genes were defined as ethanol-responsive (Additional file 6). Only one gene Cthe_3078 (*olpB*) was down-regulated, although this result was not reflected by proteomics at 120 min time point (Additional file 5). Five ethanol-induced genes were Cthe_2811 (ManA), Cthe_0745 (CelW), Cthe_0274 (CelP), Cthe_0246 (carbohydrate binding family 6 protein, putative pectinase), and Cthe_2950 (pectate lyase), which were up-regulated at earlier stages and had no or few peptides detected following ethanol treatment (Additional file 7).

Nine genes have been reported to be related to cellulosome regulation as anti-sigma factors recently (Cthe_0059, Cthe_0260, Cthe_0267, Cthe_0316, Cthe_0404, Cthe_1273, Cthe_2119, Cthe_2522, and Cthe_2974) [42]. Cthe_2119 has been examined for its carbohydrate-binding and enzymatic performance of the GH9 module and the possibility as a biomass sensor [43]. In this study, the microarray results indicated only Cthe_2522 as an ethanol-responsive gene under the conditions used in this experiment and it was down-regulated after ethanol treatment (Additional file 3), but not detected by proteomics. However, two other proteins (Cthe_0267 and Cthe_2119) were detected by LC-MS/MS but only at low, non-quantifiable levels (Additional file 4). The cellulosome components with high gene expression levels had no significant differences between control and treatment at 120 min post-shock. Differences in gene expression were primarily from genes with low expression values.

### Ethanol effect on carbon metabolism, glycolysis and pyruvate catabolism

In our proteomic study, proteins related to glycolysis and pyruvate metabolism were among the most abundant that were detected and had the greatest number of normalized spectral counts compared to other proteins identified (Additional file 4). Forty proteins were detected that had total normalized spectral counts (the sum of two biological and two technical replicates) greater than 1,000 in either condition, and fifteen of them were related to glycolysis, pyruvate catabolism and central metabolism, including Cthe_0137 (Gap), Cthe_0138 (Pgk), Cthe_0143 (Eno), Cthe_0347 (Pfk), Cthe_0349 (Fba), Fe hydrogenases Cthe_0341-2 and Cthe_0429-30, iron-containing alcohol dehydrogenase Cthe_0394, bifunctional acetaldehyde-CoA/alcohol dehydrogenase Cthe_0423, NADP-dependent isocitrate dehydrogenase Cthe_0285, malate dehydrogenase Cthe_0344, and pyruvate flavodoxin/ferredoxin oxidoreductase complex genes Cthe_2392 and Cthe_2393 (Additional file 4).

Growth and metabolomic data indicated that the ethanol shock arrested cell growth and led to the intracellular accumulation of the substrate (cellobiose) as well as its glycolytic intermediates, i.e. glucose-6-P, fructose-6-P, and 3-P-glycerate (Additional file 2). Transcriptomic data were consistent with metabolomic results, as several genes (Cthe_0347, Cthe_0946, Cthe_2390, Cthe_2393, Cthe_3020, Cthe_3021, Cthe_3120) related to glycolysis and pyruvate metabolism were down-regulated in the ethanol treatment condition, as well as the *pta* (Cthe_1028) and *ack* (Cthe_1029) genes for acetate production (Additional file 3).

### Ethanol effect on nitrogen metabolism

Urea ABC transport components were up-regulated after ethanol treatment at the gene (Cthe_1819 to Cthe_1823) and protein levels (Cthe_1823) (Table 1, Additional files 3 and 5), which likely facilitates *C. thermocellum* urea uptake in this experiment (urea and NH_4_Cl are provided in the medium). The urease gene cluster (Cthe_1813 to Cthe_1818) was also up-regulated after ethanol stress and is likely responsible for converting accumulated urea into ammonia for anabolism (Additional file 5). There was less glutamate within the ethanol-treated cells compared to that of control cells (Additional file 2). Genes related to glutamate-specific aminoacyl-tRNA biosynthesis, such as glutamyl-tRNA synthetase, glutaminyl-tRNA synthetase, and aspartyl/glutamyl-tRNA(Asn/Gln) amidotransferase were relatively less abundant and down-regulated with the ethanol treatment (Additional file 3). Genes related to glutamate metabolism, however, were up-regulated with the ethanol treatment. For example, genes encoding the glutamine synthetase (Cthe_1539, Cthe_0196-8) were always up-regulated in ethanol-treated cells compared to control cells. Other glutamate metabolism related genes such as glutamate dehydrogenase (Cthe_0374) were also up-regulated in the ethanol treatment condition. Cthe_0374 was the most abundant transcript related to glutamate metabolism (Additional file 3). In accordance with the transcriptomic data, the proteomic data indicate that the same genes related to glutamate metabolism (Cthe_0196, Cthe_0197, Cthe_0198, Cthe_0200 & Cthe_1539) were also up-regulated after treatment (Table 1, Additional file 3).

## Discussion

### Ethanol stress inhibits glycolysis

After ethanol treatment, culture growth was slowed but not completely halted under pH controlled conditions (Figure 1). Nutrient exhaustion or a decrease in pH from acid formation have been suggested as more important factors in growth inhibition than direct end-product accumulation [44]. Rydzak et al. (2011) have shown that changes in enzyme activities in response to exogenous end product additions (including ethanol) did not correlate with final end-product yields and suggested that end-product yields may be governed by thermodynamic considerations.

The rate of acetate production, whose biosynthesis generates an ATP [11], was reduced by about one half in the treated cultures and having less ATP available likely contributed to lower growth rates for the treated cultures (Figure 1). However, the final concentration of acetate was not dramatically different between the treated and control fermentations at the end of the experiment (Figure 1). Likewise, final net ethanol concentrations (~ 0.41 or 0.35 g/L for control/treatment fermentations, respectively) were similar between conditions and in keeping with prior studies [44,45].

In this study, we examined the impact of ethanol addition on metabolism. Glycolysis and pyruvate catabolism were arrested after ethanol treatment, which led to the accumulation of the substrate cellobiose and glycolytic intermediates within the ethanol-treated cells (Additional file 2) and end-product formation was linked to cellobiose consumption, as has been observed previously [45,46]. Enzymes involved in glycolysis and pyruvate catabolism were among the most abundant proteins identified in both the control and ethanol-treated cells (Additional file 4), indicative of their pivotal role in cellular metabolism [46]. In this study, we assayed the relative abundances and correlations of many important *C. thermocellum* genes, proteins, and metabolites for the first time. Glyceraldehyde-3-phosphate dehydrogenase (Cthe_0137), phosphoglycerate kinase (Cthe_0138), fructose-1,6-bisphosphate aldolase (Cthe_0349), and bifunctional acetaldehyde-CoA/alcohol dehydrogenase (Cthe_0423) had the greatest number of normalized spectral counts relative to other detected proteins. Though highly abundant, these enzymes did not show large, significant differences and many showed no significant differences between the ethanol treatment and control samples at 120 min post ethanol stress (Table 1). The overall correlation (*R*^*2*^ = 0.34) between array and proteomics was in keeping with previous studies for different microorganisms [47] (Table 1, Additional file 8).

Several key genes involved in the glycolysis pathway were down-regulated upon addition of ethanol, although their values were less than the 2-fold cut-off value and were thus not included in the significantly differentially expressed ethanol-responsive gene list (Additional file 3). We did not investigate allosteric regulation in this study. Inhibition of glycolysis would lead to less reducing power (in the form of NADH) being available for downstream electron transport and ethanol production. The decrease in ethanol production and reduced electron flux may generate insufficient NAD^+^ to ensure normal cellular metabolism. Cells may overcome this deficiency with lactate biosynthesis generating more NAD^+^, which may also explain the increase of lactate production from 0.014 ± 0.003 g/L in control cells to 0.035 ± 0.016 g/L in ethanol-treated cells to in this study.

### Hydrogenases and energy metabolism

Carbon and nitrogen metabolism were affected by ethanol stress and proteins related to electron flow were also impacted. There is surprising diversity amongst clostridial hydrogenases, and a better understanding of hydrogenases will facilitate their application and manipulation for bioenergy requirements [48]. *C. thermocellum* ATCC 27405 contains six hydrogenases, a NiFe energy converting hydrogenase and five FeFe containing enzymes [48]. Transcription of NiFe and FeFe hydrogenases in *C. thermocellum* has been confirmed by RT-PCR [49].

Most FeFe hydrogenases were up-regulated in response to ethanol shock, except for a hydrogenase encoded by Cthe_0340-2 (Additional file 8). In contrast to hydrogenase Cthe_0335, other FeFe hydrogenases were growth-phase dependent and down-regulated in the stationary phase, which is consistent with earlier data [49]. Interestingly, the Fe only hydrogenase subunit Cthe_3003 contains a glutamate synthase binding domain. Cthe_3004 is a putative ferredoxin containing a NADPH binding domain and a pyridine nucleotide-disulphide oxidoreductase binding domain [48,49], and was induced with ethanol treatment. The hydrogenase subunits (Cthe_3003 and Cthe_3004) and the NADPH-dependent glutamate synthase Cthe_0198 showed similar profiles, being all up-regulated by ethanol and with lower abundance within cells under the conditions used in this study (Additional file 8).

The multi-subunit membrane-associated NiFe hydrogenase complex (EchA-F) is encoded by the Cthe_3019-24 genes, which were repressed by the ethanol treatment dramatically and the response occurred rapidly after ethanol shock (Additional file 8). The differential expression of these hydrogenase genes may indicate that they play a role in rebalancing the cells redox state after ethanol stress. Except for subunit EchE (Cthe_3020) that had only a few spectra assigned, no peptides were detected for the remaining five subunits in 120 min post-shock (Additional file 8). The hydrogenase assembly and maturation genes (Cthe_3013-8) are adjacent to NiFe hydrogenase and form a putative operon with Cthe_3019 (4Fe-4S ferredoxin iron-sulfur binding domain-containing protein). The Cthe_3013-8 genes were also down-regulated in response to ethanol shock, and except for hydrogenase accessory protein HypB (Cthe_3017) with 10 spectra detected, other hydrogenase accessory proteins were not detected in the proteomic studies in 120 min post-shock (Additional file 8). The lack of spectra may indicate a limitation in detection of these membrane proteins.

Recently, a genome-scale metabolic analysis of *C. thermocellum* for bioethanol production has been reported [50], and the *in silico* metabolic modeling results indicated that the deletion of nine non-essential genes affecting redox balance or acetate production could possibly increase ethanol production. Of these, seven genes are involved in energy metabolism and redox balance, including the potential operon RnfCDGEAB and hydrogenase Cthe_3003 [50]. In this study, the Rnf type membrane-associated NADH:ubiqinone oxidoreductase RnfCDGEAB encoded by a potential operon (Cthe_2430-5) [49] was also down-regulated with ethanol treatment and the response was instantaneous (Additional file 8). Except for subunit A (RnfA), all other subunits were detected by LC-MS/MS. An association of RnfCDGEAB with the NiFe hydrogenase for energy generation is further suggested by their co-expression patterns in this study. Differential expression for genes such as Cthe_1559 (Cystathionine gamma-synthase) and Cthe_1560 (Pyridoxal-5'-phosphate-dependent enzyme, beta subunit) may indicate pathways to rebalance energy and requirements for new amino acids although further studies are required (Additional file 3).

### Nitrogen assimilation and metabolism

Bacteria are able to use a range of different nitrogen compounds, depending upon environmental availability and cellular requirements. In the case of industrial, solvent-producing *Clostridium* species, the source of nitrogen affects solvent yield and when nitrogen deficient molasses replaced historical corn mash as a feed stock, nitrogenous compounds had to be added to the fermentation [51].

In this study, genes involved in nitrogen metabolism were among the most significantly up-regulated after ethanol treatment. The majority of urea ABC transport (Cthe_1819-1823) and urease (Cthe_1812-1818) genes were significantly up-regulated immediately post-shock and remained high, relative to the control, at all remaining points in the time course (Additional file 6). *Staphylococci aureus* urease genes have also been reported to show greater expression following ethanol stress and play a key role in pathogenesis [52]. Here, urea was provided as a major nitrogen source in the medium likely triggered the urea ABC transport and urease gene expression upon stress conditions. The high levels of differential gene expression for genes involved in nitrogen metabolism may indicate a requirement for biosynthesis of new amino acids. Media composition and engineering urease genes in *Thermoanaerobacterium saccharolyticum* were reported to be among the most important parameters for strain improvement [53], which may point to future directions for *C. thermocellum* improvements for biotechnology.

Glutamate was depleted in ethanol-treated cells soon after ethanol addition and glutamine synthetases (Cthe_0196, Cthe_1539), both at the gene and protein levels (Table 1), were up-regulated following ethanol stress. In contrast, glutamyl-tRNA and glutiminyl-tRNA synthetase genes were down-regulated. LC-MS/MS proteomics identified several proteins related to glutamine metabolism, for example Cthe_1867, Cthe_1868, Cthe_0949, Cthe_0950, Cthe_1162 and Cthe_1249, however only carbamoyl-phosphate synthase large chain 1 (Cthe_0949) was up-regulated by ethanol treatment. It is possible that a major fraction of glutamine may flow into the biosynthesis of carbamoyl-P, leading to changes in pyrimidine metabolism as well as arginine and proline metabolism. Furthermore, the aspartate carbamoyltransferase gene (Cthe_0953) was also induced with the ethanol treatment, with gene expression up-regulated in the early time point of 12 min post-shock and then down-regulated (Additional file 3). *C. thermocellum* ethanol tolerant mutant strains have been found with nonsynonymous SNPs in Cthe_0953 [22]. In this study, proteomics data indicated an increased abundance of Cthe_0953 (and proteins encoded by adjacent genes) within the ethanol-treated cells, which may also lead to synthesis of building blocks through aspartate (Asp). Urea cycle intermediates and urea are known to react with ethanol and form inhibitory compounds [22]. Cthe_0556 (asparagine synthase, glutamine-hydrolyzing) and Cthe_0069 (Aspartate--ammonia ligase) had similar levels between control and treatment. Up-regulation of the Cthe_3158, Cthe_2874 and Cthe_0755 genes and the possible absence of asparagine (Asn) synthesis from aspartate therefore may provide the aspartate substrate for the down-stream N-carbamoyl-L-aspartate synthesis. The extent to which *C. thermocellum* is rebalancing its metabolism in response to ethanol specifically or as a general stress response can likely be answered best by examining other *C. thermocellum* stress responses.

## Conclusion

The inhibition of glycolysis and pyruvate catabolism, together with the induction of nitrogen uptake and metabolic genes indicates that *C. thermocellum* redirects carbon and nitrogen flux to generate required building blocks to overcome cell arrest and reestablish growth. Further work is needed to examine the cross talk between carbon and nitrogen metabolism and access to newly developed *C. thermocellum* genetic systems will facilitate these analyses in the future. These data will assist others as they begin to examine respective transcript and protein levels in *C. thermocellum* metabolic engineering, considering useful promoters as an example. A greater number of systematic and more detailed studies that examine physiology at various “omics” levels are required to realize the potential application of synthetic biology approaches in important microorganisms such as *C. thermocellum*.

## Competing interests

The authors declare that they have no competing interests.

## Authors' contributions

SY conceived and designed the study, conducted the fermentations, analyzed microarray data, interpreted all data and wrote the manuscript; RJG generated and analyzed proteomics data and wrote the manuscript; LD generated microarray data, qPCR data and assisted with fermentations; ZKY assisted with RNA extractions; NLE generated metabolomics data; TJT analyzed metabolomics data and wrote the manuscript; RLH analyzed proteomics data and wrote the manuscript; SDB conceived and designed the study, analyzed the data and wrote the manuscript. All authors read and approved the final manuscript.

## Supplementary Material

Additional file 1 ** qPCR Primers used to confirm transcriptomic results.** DNA sequences for oligonucleotides used in this study. Click here for file

Additional file 2 ** Metabolomic profiling of *C. thermocellum *ATCC27405 with ethanol treatment at different time points post ethanol-shock compared to that of control.** The relative metabolite fold-change responses of ethanol treatment compared to that of control are shown at different time points. Ethanol supplemented for ethanol treatment was 3.95 g/L (equal to 0.5% [v/v]) at mid-exponential phase. The control condition was without ethanol supplementation. Click here for file

Additional file 3 ** All 3047 differently expressed genes.** Genes significantly differentially expressed with ratio greater than 2-fold within at least one comparison of ethanol treatment versus control and time course studies of both ethanol-treated and control cells. Click here for file

Additional file 4 ** Proteins with peptide hits identified from a shot-gun proteomics study for both ethanol-treated and control cells at 120 min post ethanol-shock.** Raw and normalized spectral counts for peptides identified 1317 proteins. Relative differences between conditions and significance values are shown. Click here for file

Additional file 5 ** Significantly differentially expressed with at least a 2-fold change for ethanol-treated cells compared to control cells at same time points.** A total of 641 genes were differentially expressed for time point comparisons at 2, 12, 30, 60, 120, and 240 min. Click here for file

Additional file 6 ** Potential ethanol-responsive genes.** A total of 158 ethanol-responsive genes were identified by treatment versus control comparisons and treatment time series comparisons that where control time series comparisons were not responsive. Click here for file

Additional file 7 ** Comparison among proteomics, transcriptomics and literature results for 70 genes related to cellulosomes.** Examination of trends across different omics datasets for cellulosome components. Click here for file

Additional file 8 ** Comparison among proteomics, transcriptomics and literature results for 17 genes related to hydrogenases.** Examination of trends across different omics datasets for hydrogenase components. Click here for file
