# Supplementary material for: Clostridium thermocellum ATCC27405 transcriptomic, metabolomic and proteomic profiles after ethanol stress
Source: BMC Genomics. 2012 Jul 23;13:336. doi: 10.1186/1471-2164-13-336 (PMC3478167; doi:10.1186/1471-2164-13-336)
Supplement: Additional file 1 — qPCR Primers used to confirm transcriptomic results. DNA sequences for oligonucleotides used in this study. [file 1471-2164-13-336-S1.docx]

**Additional file 1. qPCR Primers used to confirm transcriptomic results.**

| **Primer ID** | **Sequence (written 5' - 3')** |
| --- | --- |
| Cthe_0197_F | tgacggtgatgtggctaaag |
| Cthe_0197_R | tcccttggtctttttcgttg |
| Cthe_0665_F | ggaattcaggtgctggatgt |
| Cthe_0665_R | gtctccttgctctgctttgc |
| Cthe_0736_F | tcaatggtcgtgcatgattt |
| Cthe_0736_R | ttcttctgccttgcctgaat |
| Cthe_0745_F | atcccacagcggattctatg |
| Cthe_0745_R | aggattcggctttgtcaaga |
| Cthe_1028_F | caggtatcggcgaaaacaat |
| Cthe_1028_R | cgcttctttgcctttcactt |
| Cthe_1539_F | ctcattttcctggtggctgt |
| Cthe_1539_R | ggctccaagtctgtggtcat |
| Cthe_1565_F | ggctgtgctacctcaaaagc |
| Cthe_1565_R | ttcatgcagacaggaagcac |
| Cthe_2301_F | tgttgattgagctggacagg |
| Cthe_2301_R | agcgcatttacacgcttttt |
| Cthe_2336_F | tcagtttgcgggtgatgata |
| Cthe_2336_R | accgtgcaagttcaatcctc |
| Cthe_2435_F | aggcgtgcaagcaaaagtat |
| Cthe_2435_R | acgcaaccgtaagaacatcc |
| Cthe_3016_F | tccctatgacaggcacaaca |
| Cthe_3016_R | atatggtttgggcgtgaaag |
