# Supplementary material for: Clostridium thermocellum ATCC27405 transcriptomic, metabolomic and proteomic profiles after ethanol stress
Source: BMC Genomics. 2012 Jul 23;13:336. doi: 10.1186/1471-2164-13-336 (PMC3478167; doi:10.1186/1471-2164-13-336)
Supplement: Additional file 2 — Metabolomic profiling of C. thermocellum ATCC27405 with ethanol treatment at different time points post ethanol-shock compared to that of control. The relative metabolite fold-change responses of ethanol treatment compared to that of control are shown at different time points. Ethanol supplemented for ethanol treatment was 3.95 g/L (equal to 0.5% [v/v]) at mid-exponential phase. The control condition was without ethanol supplementation. [file 1471-2164-13-336-S2.doc]

**Additional file 2. Metabolomic profiling of *C. thermocellum*** ATCC27405 with ethanol treatment at different time points post ethanol-shock compared to that of control.

|  |  |  | |  | Time (min) | |  |  |
| --- | --- | --- | --- | --- | --- | --- | --- | --- |
| Metabolite | 2 | | 5 | 12 | 30 | 60 | 120 | 240 |
| cellobiose | 1.95 | | 1.08 | 2.07 | 1.49 | 1.82 | 2.87 | 4.35* |
| glycerol | 2.92 | | 1.17 | 2.47 | 1.83 | 2.27 | 2.52 | 3.67 |
| phenylalanine | 2.57 | | 2.09 | 2.32 | 1.46 | 1.90 | 1.68 | 2.81** |
| carbamyl aspartic acid | 1.36 | | 1.26 | 1.23 | 0.98 | 0.99 | 1.69 | 1.95 |
| fructose-6-P | 1.68 | | 3.96 | 2.46 | 2.28* | 1.95** | 1.72** | 1.80* |
| glucose-6-P | 1.56 | | 6.73 | 2.80 | 2.45* | 2.23** | 1.68** | 1.80* |
| heptadecanoic acid | 0.82 | | 2.09 | 1.01 | 0.92 | 1.12 | 1.21 | 1.79 |
| phosphate | 1.58 | | 1.13 | 1.64 | 1.11 | 1.16 | 1.29 | 1.68 |
| palmitic acid | 0.99 | | 1.37 | 0.96 | 0.77 | 0.95 | 1.07 | 1.53 |
| glucose-1-P | 1.41 | | 2.10 | 1.65 | 0.83 | 0.86 | 1.18 | 1.52 |
| glutamine | 0.75 | | 0.51 | 0.74 | 0.42 | 0.60 | 1.13 | 1.40 |
| urea | 1.46 | | 1.12 | 1.53 | 0.98 | 1.05 | 1.20 | 1.32 |
| 1,6-anhydroglucose | 1.02 | | 1.22 | 1.31 | 0.76 | 1.01 | 1.05 | 1.31 |
| t-aconitic acid | 1.51 | | 1.17 | 1.15 | 0.71 | 0.79 | 0.94 | 1.27 |
| cysteine | 1.63 | | 1.44 | 1.32 | 0.80 | 0.84 | 1.00 | 1.27 |
| 3-phosphoglyceric acid | 1.44 | | 1.39 | 1.09 | 1.40 | 2.03 | 1.34 | 1.22** |
| glycerol-1/3-P | 0.98 | | 1.48 | 1.15 | 0.84 | 0.92 | 0.83 | 1.16 |
| stearic acid | 1.41 | | 1.63 | 1.18 | 0.78 | 0.89 | 0.82 | 1.14 |
| 9.65 174 114 100 276 | 1.42 | | 1.24 | 1.51 | 1.19 | 1.22 | 1.26* | 1.07 |
| glutamic acid | 0.65 | | 0.43 | 0.34* | 0.19** | 0.27 | 0.48 | 0.76 |
| proline | 0.83 | | 0.94 | 0.79 | 0.81 | 0.75 | 0.48 | 0.66 |

The relative metabolite fold-change responses of ethanol treatment compared to that of control are shown at different time points.

Ethanol supplemented for ethanol treatment was 3.95 g/L (equal to 0.5% [v/v]) at mid-exponential phase. The control condition was without ethanol supplementation.

* Denotes statistical significance at P<0.10.

** Denotes statistical significance at P<0.05.
